# A method of using deep learning to predict three‐dimensional dose distributions for intensity‐modulated radiotherapy of rectal cancer

**DOI:** 10.1002/acm2.12849

**Published:** 2020-04-13

**Authors:** Jieping Zhou, Zhao Peng, Yuchen Song, Yankui Chang, Xi Pei, Liusi Sheng, X. George Xu

**Affiliations:** ^1^ Department of Radiation Oncology The First Affiliated Hospital of USTC, Division of Life Sciences and Medicine University of Science and Technology of China Hefei Anhui China; ^2^ Department of Engineering and Applied Physics School of Physics University of Science and Technology of China Hefei Anhui China; ^3^ National Synchrotron Radiation Laboratory University of Science and Technology of China Hefei Anhui China; ^4^ Anhui Wisdom Technology Company Limited Hefei Anhui China; ^5^ Nuclear and Biomedical Engineering Rensselaer Polytechnic Institute Troy NY USA

**Keywords:** 3D deep learning architecture, dose prediction, IMRT, rectal cancer

## Abstract

**Purpose:**

To develop and test a three‐dimensional (3D) deep learning model for predicting 3D voxel‐wise dose distributions for intensity‐modulated radiotherapy (IMRT).

**Methods:**

A total of 122 postoperative rectal cancer cases treated by IMRT were considered in the study, of which 100 cases were randomly selected as the training–validating set and the remaining as the testing set. A 3D deep learning model named 3D U‐Res‐Net_B was constructed to predict 3D dose distributions. Eight types of 3D matrices from CT images, contoured structures, and beam configurations were fed into the independent input channel, respectively, and the 3D matrix of dose distributions was taken as the output to train the 3D model. The obtained 3D model was used to predict new 3D dose distributions. The predicted accuracy was evaluated in two aspects: (a) The dice similarity coefficients (DSCs) of different isodose volumes, the average dose difference of all voxels within the body, and 3%/5 mm global gamma passing rates of organs at risks (OARs) and planned target volume (PTV) were used to address the spatial correspondence between predicted and clinical delivered 3D dose distributions; (b) The dosimetric index (DI) including homogeneity index, conformity index, V_50_, V_45_ for PTV and OARs between predicted and clinical truth were statistically analyzed with the paired‐samples t test. The model was also compared with 3D U‐Net and the same architecture model without beam configurations input (named as 3D U‐Res‐Net_O).

**Results:**

The 3D U‐Res‐Net_B model predicted 3D dose distributions accurately. For the 22 testing cases, the average prediction bias ranged from −1.94% to 1.58%, and the overall mean absolute errors (MAEs) was 3.92 ± 4.16%; there was no statistically significant difference for nearly all DIs. The model had a DSCs value above 0.9 for most isodose volumes, and global 3D gamma passing rates varying from 0.81 to 0.90 for PTV and OARs, clearly outperforming 3D U‐Res‐Net_O and being slightly superior to 3D U‐Net.

**Conclusions:**

This study developed a more general deep learning model by considering beam configurations input and achieved an accurate 3D voxel‐wise dose prediction for rectal cancer treated by IMRT, a potentially easier clinical implementation for more comprehensive automatic planning.

## 1 INTRODUCTION

Intensity‐modulated radiotherapy (IMRT) has been widely used for treating many cancers.^1^, ^2^, ^3^ The design process of the inverse IMRT treatment plan is very complex due to a large number of parameters related in particularly to the optimized objective functions.^4^, ^5^ Since the patient‐specific dose distributions are unknown, the objective functions are usually defined by the treatment planner according to standard clinical protocols. Such protocols are based on population‐average data without considering individualized dose information. In designing the treatment plan, the planner has to make adjustment repeatedly in a trial‐and‐error manner until the dose distributions are deemed to meet the clinical specific criteria. However, the experience, skill, and time available to the planners vary drastically among different medical centers, resulting in variable treatment plan qualities.^6^, ^7^

Clearly, the ability to accurately predict dose distributions will bring the opportunity to improve the quality of the treatment plans in busy Chinese clinics, including the First Affiliated Hospital of USTC (The University of Science and Technology of China, Hefei, China) where more than 300 patients are treated each day on the average.

Researchers have devoted a considerable amount of efforts in achievement the dose prediction as part of the treatment planning process. The most widely reported approach is the so‐called knowledge‐based planning (KBP) which builds a geometry‐dosimetry correlation prediction model based on prior patient databases of high‐quality treatment plans^8^, ^9^, ^10^, ^11^, ^12^, ^13^, ^14^, ^15^, ^16^, ^17^, ^18^, ^19^ and this approach is currently available in the commercial software, RapidPlan (Varian Medical Systems, Palo Alto, CA).^20^ These KBP methods have been shown to improve the plan quality, consistency, and efficiency. However, the methods have two limitations. First, most of these predicted dose distributions are expressed as one‐dimensional dose‐volume histogram (DVH) or zero‐dimensional dosimetric endpoints that may correspond to nonunique 3D dose distributions. As a result, the final plans with acceptable DVH objectives can still contain unacceptable dose distributions. When that situation occurs, the planner needs to manually add planning‐auxiliary contours and reoptimize plan in order to refine the spatial dose distributions. Second, these methods usually rely on the extraction of handcrafted features, such as overlapping volume histogram,^21^, ^22^ and distance to target histogram.^23^, ^24^ However, handcrafted features on the patient plan do not cover all inherent structure characteristics, so the quality of dose distributions prediction cannot be easily improved. Meanwhile, the extraction process of handcrafted features is complex and tedious, making it difficult to implement in a busy clinic like ours. For these reasons, it is attractive to develop an algorithm that can automatically extract features from the patient contoured structures for the purposes of achieving more accurate and effective prediction of 3D dose distributions.

The advent of advanced computer hardware and deep learning (DL) tools has brought breakthroughs in medical informatics in recent years.^25^, ^26^ The convolutional neural network (CNN), in particular, is the most common DL‐based tool for image analysis.^27^, ^28^ Convolutional neural network models can automatically extract hierarchical features from the image data and achieve the end‐to‐end prediction without omissions and tediousness of many manual processes. To date, CNN‐based methods have been used to achieve excellent performance in radiotherapy workflows including automatic segmentation,^29^, ^30^, ^31^ deformable registration,^32^, ^33^ and synthetic computed tomography (CT) generation from magnetic resonance (MR) images.^34^

In this paper, we proposed a 3D CNN model (called 3D U‐Res‐Net_B) based on 3D U‐Net^35^ and Residual network^36^ to achieve voxel‐wise dose prediction for postoperative rectal cancer patients treated by IMRT. First, eight types of 3D matrices were extracted from the CT images, contoured structures and beam configurations. The matrices were then put into different input channels of the 3D U‐Res‐Net_B model, and the 3D matrix of clinically delivered dose distributions was used as the output. The 3D U‐Res‐Net_B can learn multiple‐scale and multiple‐level features of anatomy and beam configurations, and then map these features to 3D dose distributions.

In next sections, we introduce the patient data processing methods in Section 2.A, the 3D CNN model and training methods in Section 2.B, and the evaluation methods in Section 2.C. Then, we present experimental results in Section 3 and discuss the experimental results and related researches in Section 4.

## 2 MATERIALS AND METHODS

### 2.1 Patient database and data processing

A total of 122 postoperative rectal cancer patients undergoing IMRT between the years 2015 and 2018 were enrolled in this study, of which 100 cases were randomly selected and divided into the training and validation sets with a ratio of 4:1. The remaining 22 cases were used to test the model. The study protocol was approved by the review board of our institution. Patients were immobilized in a vacuum bag in the supine position, the bladder was emptied and then filled with 500 mL of water 1 h before the enhanced CT scanning was performed on a GE CT590 simulated localization machine (GE Healthcare, Waukesha, USA). The scanning range was from the lower edge of the L‐2 vertebra to 5 cm below the ischial tubercle with a slice thickness of 5 mm. The images were reconstructed to 2.5 mm and transmitted to the Pinnacle^3^ treatment planning system (Philips Radiation Oncology Systems, Fitchburg, WI, USA). The clinical target volume (CTV) and organs at risk (OARs) were delineated and checked by radiation oncologists, and a margin of 7 mm was applied around CTV to create the planned target volume (PTV) in consideration of the organ motion and positioning uncertainties. The IMRT plans were designed to deliver a prescription dose of 50 Gy to the PTV using 6‐MV, five to seven coplanar beams in the “step and shoot” mode, and direct machine parameters optimization (DMPO) technique.

**Fig. 1:**
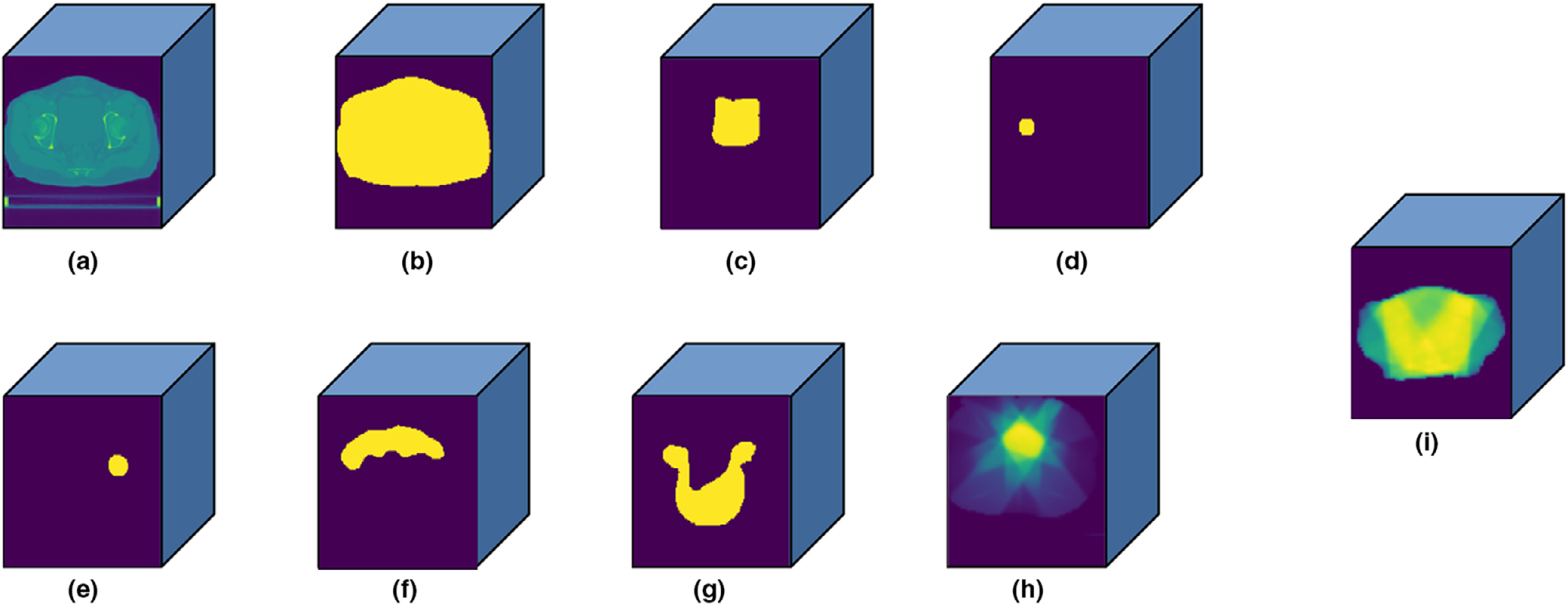
Three‐dimensional (3D) matrix (128 × 128 × 128) of (a) computed tomography images; (b) body; (c) bladder; (d) right femoral head; (e) left femoral head; (f) small intestine; (g) planned target volume; (h) beam configurations, and (i) 3D dose distribution.

The original CT images, contoured structures, beam configurations information, and delivery dose of the IMRT plan were exported from Pinnacle system and then converted to 3D matrices using a developed in‐house python software program, as shown in Fig. 1. The CT images were extracted as a 3D CT matrix where the CT values were first truncated to range between −200 and 300 HU and then normalized to the range of −1 to 1. Every patient has six contoured structures including the PTV, bladder, small intestine, left femoral head, right femoral head, and body, each being converted to a respective 3D matrix of binary mask. Three‐dimensional matrix of beam configurations was represented by cumulative dose distributions of 3D conformal radiotherapy (3D‐CRT). The aperture of the PTV projection in beam's eye view, with an isotropic margin of 5mm, was generated for each beam with the same weight proportion, and using the same beam orientation as IMRT. Convolution superposition (CS) dose calculation method was employed to compute the nonmodulated 3D dose of each beam. Final dose distributions were given by summing up the doses of all beams. The 3D dose matrix was derived from the delivered dose distribution of the IMRT plan. All 3D matrices were resampled to the same resolution of 2.5 × 2.5 × 2.5 mm^3^. To reduce the unimportant background area, these 3D matrices were cropped into the size of 128 × 128 × 128.

### 2.2 3D U‐Res‐Net_B architecture and model training

**Fig. 2:**
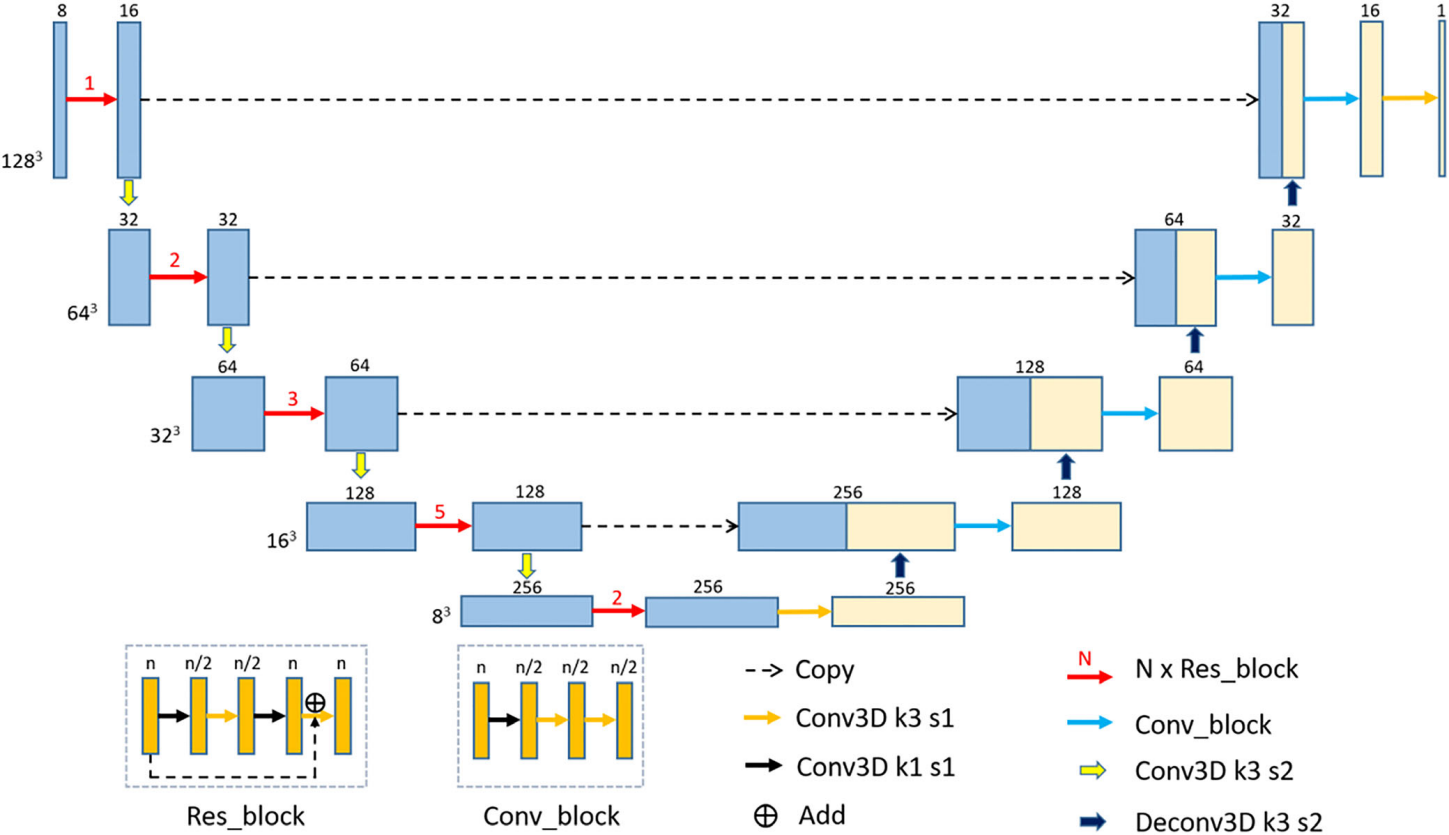
The proposed three‐dimensional (3D) U‐Res‐Net_B architecture. Each box denotes the 3D feature maps. The number and the size of feature maps are showed on the top and the left of the box, respectively. The arrows indicate different operations, the red numbers above the red arrows denote the number of Res_block, and the k and s after the Conv3D denote the kernel size and the stride of the convolution layer.

The adopted network in this study is based on a 3D U‐Net and Residual network, named as 3D U‐Res‐Net_B. As shown in Fig. 2, our proposed network consists of an encoder which extracts image features and a decoder which performs a voxel‐wise regression to achieve dose prediction. The encoder contains five encoding modules, each of which is stacked by different numbers of Res_block. The Res_block is made up of four convolution layers where the size of the convolutional kernel is 1 × 1 × 1, 3 × 3 × 3, 1 × 1 × 1, and 3 × 3 × 3, respectively. At the end of the first four encoding modules, a convolution layer with the kernel of 3 × 3 × 3 and the stride of 2 × 2 × 2 is used for the downsampling. Each convolution layer in the encoder is followed by a batch normalization and a rectified linear unit (ReLU) operation. The decoder contains five decoding modules, each including a Conv_block except the first module which contains only one 3 × 3 × 3 convolution layer. The Conv_block is made up of three convolution layers where the size of the convolutional kernel is 1 × 1 × 1, 3 × 3 × 3, and 3 × 3 × 3, respectively. At the end of the first four decoding modules, a deconvolution layer with the kernel of 3 × 3 × 3 and the stride of 2 × 2 × 2 is used for the upsampling. Each convolution layer in the decoder is followed by a ReLU operation. Four dashed arrow lines in the figure indicate four conveying paths that copy and reuse early feature maps as the input to later layers having the same feature map size by using a concatenation operation. Finally, a 3 × 3 × 3 convolution layer followed by a ReLU operation is used to predict the final dose maps.

The inputs of the 3D U‐Res‐Net_B model are eight channels of 3D matrix in the form of 128 × 128 × 128 × 8, the output is the dose matrix with the shape of 128 × 128 × 128 × 1. The delivered clinical dose distribution was regarded as ground truth. The mean squared error between the predicted and clinical delivered dose for each patient was selected as the loss function. The Adam optimization algorithm was used to minimize the loss function and the batch size was set to be 1 due to the limitation of GPU memory. In the training stage, the weights of the network are initialized randomly from scratch. At each epoch, the validation loss is monitored and the weight is updated. The learning rate starts from 0.0005 and is divided by 10 when the validation loss do not significantly become smaller in successive 10 epochs. The training process stops automatically when the validation loss do not become smaller in successive 30 epochs.

**Fig. 3:**
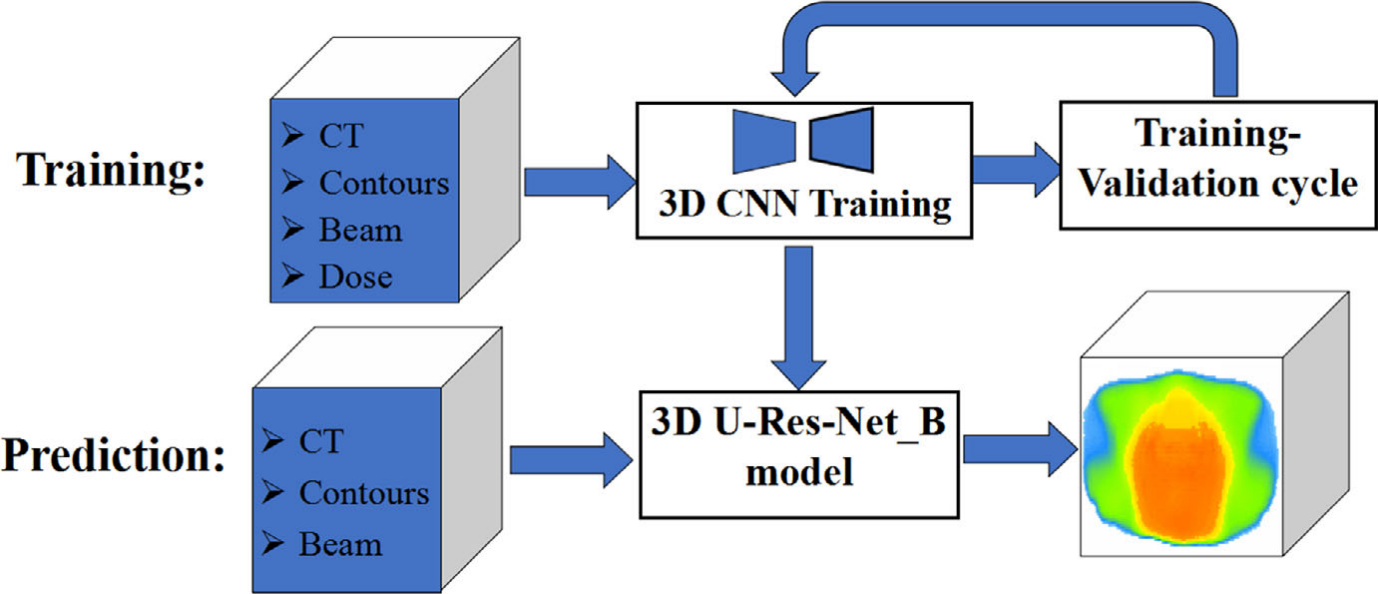
Flowchart showing training and prediction of three‐dimensional (3D) dose prediction based on the 3D U‐Res‐Net_B model.

The Python deep learning library Keras^37^ with TensorFlow^38^ as backend was employed to achieve the 3D U‐Res‐Net_B architecture. An Nvidia Geforce RTX 2080 GPU card with 8G memories was used to train the model. Once the model has been fully trained, it takes only a few seconds to predict 3D dose distribution for a new case. An overview of the training and predicting process is illustrated in Fig. 3.

### 2.3 Prediction evaluation

To evaluate the performance of the proposed 3D U‐Res‐Net_B model, the 3D dose distributions, and DVH parameters of OARs and PTV were compared between the prediction and clinical truth.

For 3D dose distributions, voxel‐wise dose difference was evaluated using $\delta D=D_{c}-D_{p}$, where D_c_ and D_p_ denote the clinical and predicted dose of each voxel within the body, respectively. The mean and standard deviation of δD were calculated to evaluate the prediction bias and precision. Mean absolute errors, $\text{MAE}=\frac{1}{n}\sum_{i}^{n}\left|\delta D\right|$ were also calculated, where i stands for the voxel point and n is overall number of voxels within the body. Also, the performance of our model was evaluated using three commonly used metrics, including dice similarity coefficients (DSCs), Hausdorff distance 95% (HD_95_), and mean surface distance (MSD). Dice similarity coefficients provides spatial overlap information, while HD_95_/MSD measure boundary similarity of different isodose surfaces between prediction and corresponding clinical truth, according to the following formulas, respectively.

(1) $$\text{DSC}=\frac{2*V_{\text{iso}-p}*V_{\text{iso}-c}}{V_{\text{iso}-p}+V_{\text{iso}-c}},$$

(2) $$\text{HD}95=\max_{k95\%}\left(\min_{x\in S_{\text{iso}-p}}{∥x-S_{\text{iso}-c}∥}_{2},\min_{y\in S_{\text{iso}-c}}{∥y-S_{\text{iso}-p}∥}_{2}\right),$$

(3) $$\text{MSD}=\frac{1}{\left|S_{\text{iso}-p}\right|+\left|S_{\text{iso}-c}\right|}\left(\sum \min_{x\in S_{\text{iso}-p}}{∥x-S_{\text{iso}-c}∥}_{2}+\sum \min_{y\in S_{\text{iso}-c}}{∥y-S_{\text{iso}-p}∥}_{2}\right),$$

where V_iso‐p_ and V_iso‐c_ denote the certain isodose volume of prediction and clinical truth, respectively, and S_iso‐p_ **/**S_iso‐c_ denote boundary surface of the corresponding isodose volume; Max_K95%_ represents 95th percentile of the maximum, and |S_iso‐p_|**/**|S_iso‐c_| denote all voxel points of isodose surface. Isodose volumes are defined as 3D binary masks where the voxel is assigned 1 if the dose of a voxel is above certain dose threshold and 0 otherwise, while isodose surface is boundary of the isodose volumes. The DSC values were calculated with the dose threshold from 5 to 50 Gy with an interval of 1 Gy, and HD_95_/MSD were calculated from 20 to 50 Gy with an interval of 5 Gy.

A global 3D gamma analysis, which is used as a tool for IMRT plan dose verification, was employed to further evaluate the accuracy of voxel‐wise dose distribution prediction for OARs and PTV. Dose difference and distance‐to‐agreement criterion were set to be 3% and 5 mm, respectively, and the gamma passing rates were calculated above the threshold of 5% prescription dose.

With respect to DVH parameters, first of all, the overall DVH curves of PTV and different OARs were presented between the prediction and clinical truth. Second, the clinical interested dosimetric indexes (DI) were calculated, including the mean dose (D_mean_), D_2_, D_50_, D_98_ for PTV (here D_i_ means the dose received by i% of PTV volume), and D_mean_, V_45_, V_50_ for OARs (here V_i_ means volume fraction of OARs irradiated by i Gy); homogeneity index (HI)^39^ and conformation index (CI)^40^ for PTV were further calculated as following formula:

(4) $$\text{HI}=\frac{D_{2}-D_{98}}{D_{50}},$$

(5) $$\text{CI}=\frac{V_{\text{ref}}*V_{\text{ref}}}{V_{\text{ptv}}*V_{\text{pres}}},$$

where V_ptv_ and V_pres_ are the volume of PTV and the prescription dose region, respectively, and V_ref_ is the irradiated PTV volume of the prescription dose.

In order to further evaluate the performance of our 3D U‐Res‐Net_B model, the model was compared with 3D U‐Net^35^ on some performance metrics. In addition, the model was also compared with the 3D U‐Res‐Net_O model, not having the beam configurations input.

## 3 RESULTS

### 3.1 The performance of the 3D U‐Res‐Net_B model

#### 3.1.1 3D dose distributions

**Fig. 4:**
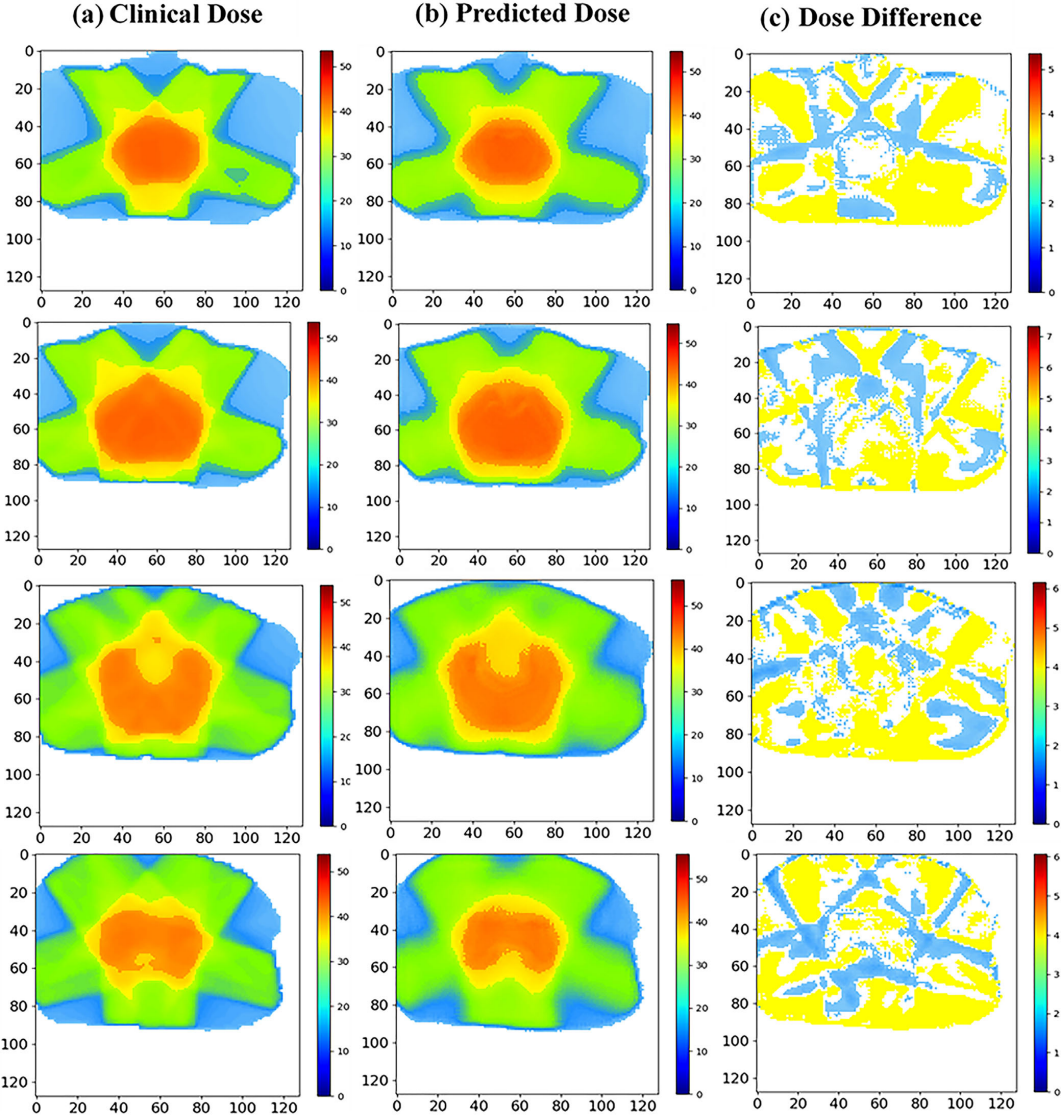
(a) clinical dose distribution, (b) predicted dose distribution, and (c) dose difference map in four trans‐axial slices of a postoperative rectal cancer case. The unit of color bar is Gy.

Figures 4(a), 4(b), and 4(c) show clinical dose distributions, corresponding predicted dose distributions, and voxel‐wise dose distribution difference map in four trans‐axial slices of a postoperative rectal cancer case. It is observed from Figs. 4(a) and 4(b) that the shape of predicted dose distributions is similar to the clinical ground truth at each of the dose levels, and the dose differences of all voxels are below 5 Gy [as can be seen in Fig. 4(c)].

**Fig. 5:**
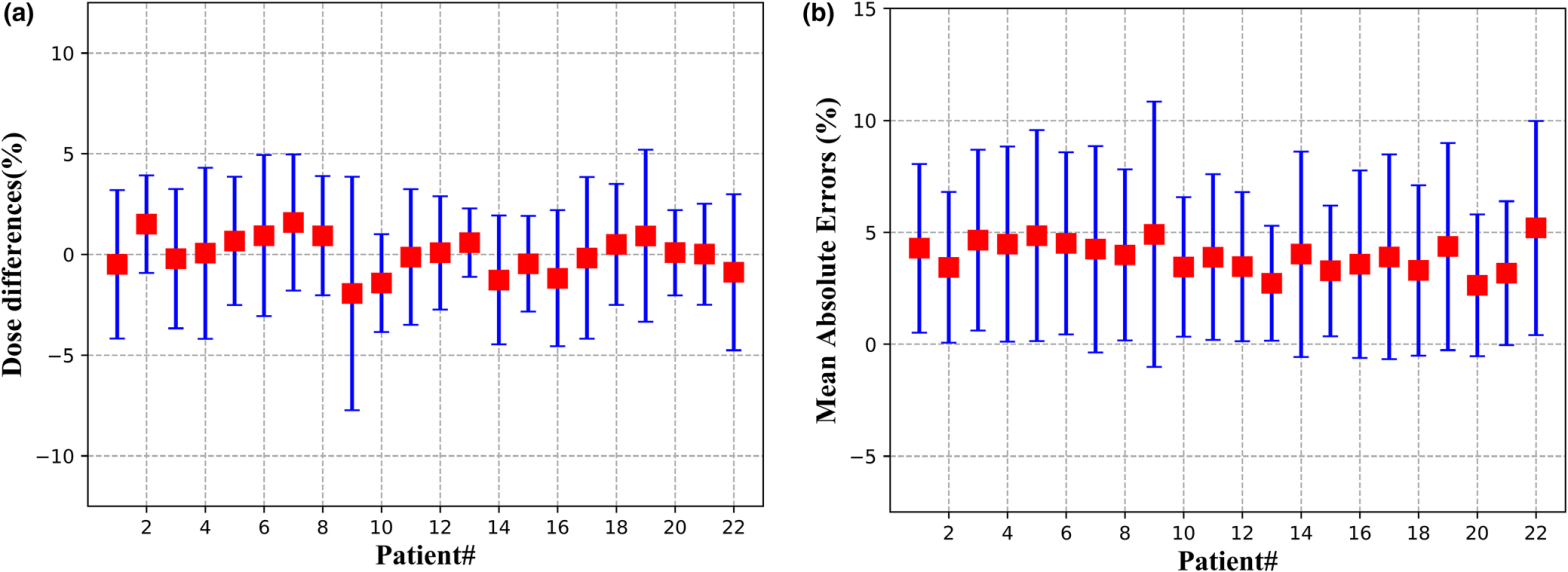
(a) Three‐dimensional dose differences; (b) mean absolute errors, including all voxels within the body for the 22 testing cases.

The mean and standard deviation of 3D dose differences of all voxels within the body for each testing patient are shown in Fig. 5(a). The red squares indicate the mean value ranging from −1.94% to 1.58%, and the blue error bars indicate the standard deviations varying from 1.70% to 5.79%. The overall averaged dose difference for all voxels of the 22 testing patients is 0.01 ± 3.28%. The MAEs including all voxels within the body for each testing patient are shown in Fig. 5(b). The largest MAE is 5.19 ± 4.78%. The smallest MAE is 2.63 ± 3.17%. The overall average MAE of the testing set is 3.92 ± 4.16%.

#### 3.1.2 Statistics of DVH dosimetric index

**Fig. 6:**
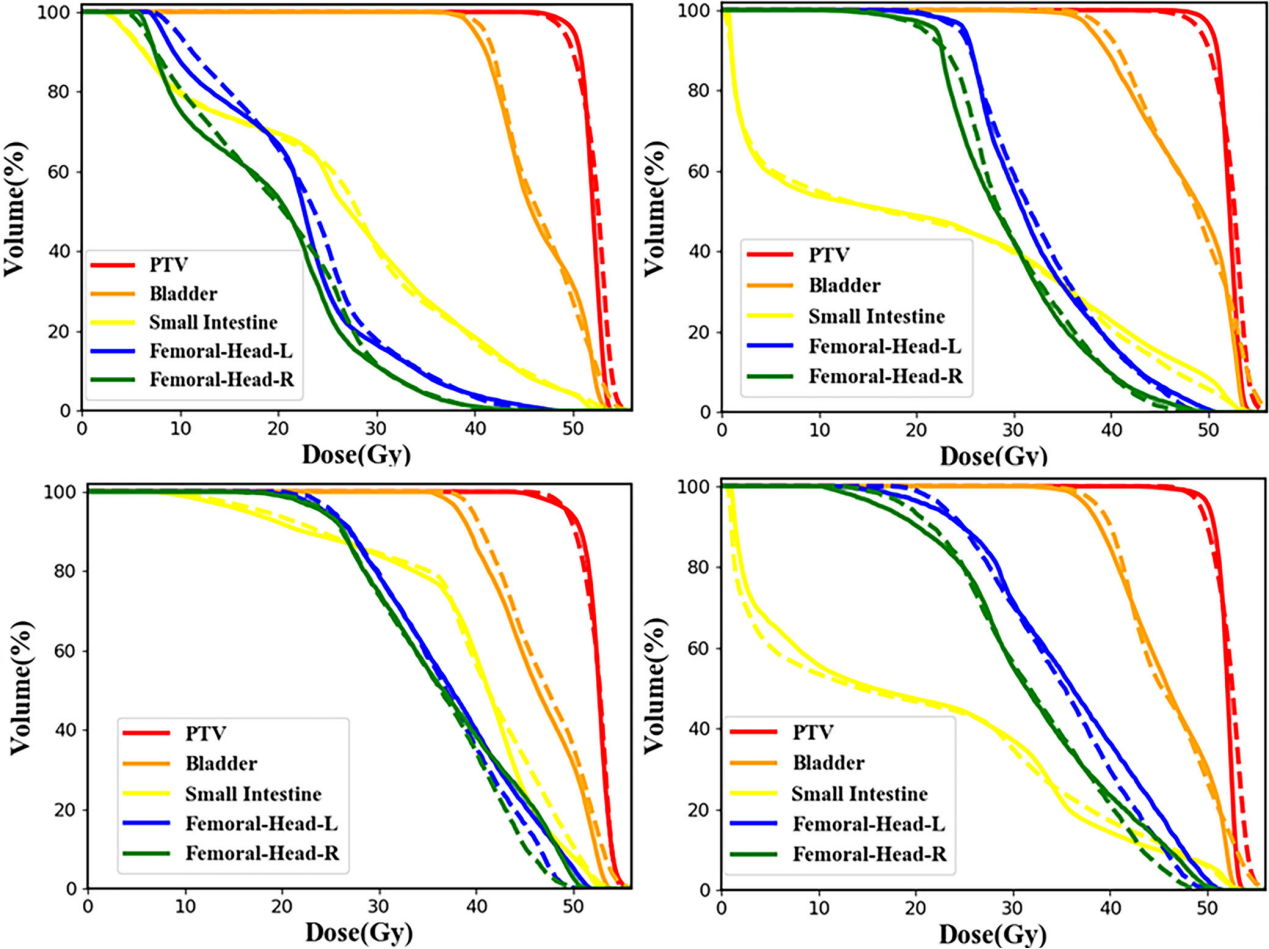
The overall dose‐volume histogram comparison of organs at risks and planned target volume between clinical (solid line) and predicted (dashed line) results for four randomly selected testing cases. Femoral‐Head‐L, left femoral head; Femoral‐Head‐R, right femoral head.

The overall DVH comparisons of PTV and OARs for four randomly selected testing patients between clinical and predicted results are presented in Fig. 6. The visual inspection indicate that the clinical and predicted DVHs of PTV and OARs have an acceptable agreement for each patient.

Table 1 shows that the mean and standard deviation of the clinically interested DI for PTV and OARs in 22 testing patients. It is found that the average absolute dose differences of D_2_, D_50_, D_98_, D_mean_ for PTV and D_mean_ for OARs are within 2.2%, and the average absolute volume differences of V_45_, V_50_ for OARs are <5%. The predicted results are comparable to clinical truth without statistical significance (*P* > 0.05), except for D_mean_ of bladder (*P* = 0.046).

**Table 1:** Mean and standard deviation of dosimetric index for planned target volume (PTV) and organs at risks (OARs).

DI	Clinical	Prediction	|bias|(%)	*P*‐value
PTV				
D98 (Gy)	48.18 ± 0.56	47.59 ± 0.65	1.82 ± 1.23	0.237
D50 (Gy)	52.29 ± 0.49	52.58 ± 0.34	0.88 ± 0.78	0.564
D2 (Gy)	54.27 ± 0.40	54.46 ± 0.61	1.52 ± 1.40	0.412
Dmean (Gy)	52.01 ± 0.40	51.98 ± 0.59	0.74 ± 0.98	0.301
HI	0.10 ± 0.03	0.12 ± 0.08	0.03 ± 0.05	0.213
CI	0.91 ± 0.02	0.90 ± 0.02	0.01 ± 0.01	0.229
Bladder				
V50 (%)	30.64 ± 10.43	32.40 ± 10.79	2.49 ± 1.58	0.120
V45 (%)	69.46 ± 16.19	68.14 ± 11.93	2.09 ± 2.27	0.453
Dmean (Gy)	46.53 ± 2.00	47.03 ± 1.64	2.00 ± 1.32	0.046
Small intestine				
V50 (%)	7.07 ± 4.63	6.53 ± 4.12	1.94 ± 1.36	0.297
V45 (%)	16.90 ± 9.04	16.89 ± 8.41	2.64 ± 2.15	0.991
Dmean (Gy)	28.32 ± 7.52	28.77 ± 6.56	1.60 ± 1.48	0.534
Left femoral head				
V50 (%)	1.21 ± 1.26	2.31 ± 3.07	1.78 ± 2.13	0.059
V45 (%)	11.42 ± 8.62	12.32 ± 12.43	4.29 ± 5.90	0.569
Dmean (Gy)	31.54 ± 5.18	31.30 ± 7.03	2.18 ± 1.03	0.774
Right femoral head				
V50 (%)	0.55 ± 0.89	0.76 ± 1.35	0.76 ± 0.11	0.489
V45 (%)	7.35 ± 6.94	7.55 ± 7.04	2.96 ± 2.05	0.874
Dmean (Gy)	29.09 ± 5.42	28.81 ± 5.55	2.08 ± 1.31	0.685

### 3.2 Comparison of model performance

**Fig. 7:**
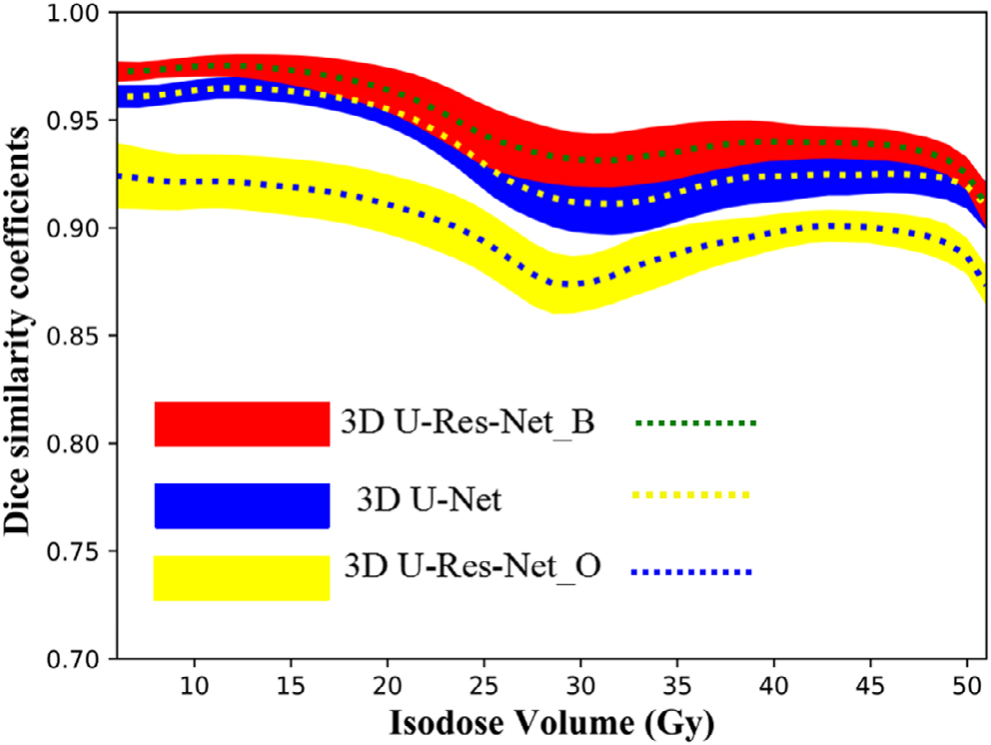
The average (dashed line) and standard deviation (color wash) of dice similarity coefficients of the isodose volumes from 5 to 50 Gy for three‐dimensional (3D) U‐Res‐Net_B, 3D U‐Net, and 3D U‐Res‐Net_O model, respectively.

The DSCs for 3D U‐Res‐Net_B, 3D U‐Net and 3D U‐Res‐Net_O are presented in Fig. 7. The 3D U‐Res‐Net_B model has a DSCs value above 0.9 for most isodose volumes, clearly outperforming 3D U‐Res‐Net_O by 5% on average, and being slightly superior than 3D U‐Net on average, with a value up to 3% higher. Additionally, a noticeable decline of DSCs at about 30 Gy isodose volumes is observed for all models, much most pronounced for 3D U‐Res‐Net_O model.

Table 2 shows Hausdorff Distance 95% (HD_95_) and Mean Surface Distance (MSD) of different isodose surfaces for 3D U‐Res‐Net_B, 3D U‐Net, and 3D U‐Res‐Net_O model. The 3D U‐Res‐Net_B reduces by around 0.4 and 0.2 cm for HD_95_ and MSD on average, respectively, with respect to 3D U‐Res‐Net_O model. Compared with 3D U‐Net model, the 3D U‐Res‐Net_B reduces by around 0.1 and 0.05 cm for HD_95_ and MSD on average, respectively. These results are consistent with those of DSCs.

**Table 2:** Statistical comparison for HD_95_ and mean surface distance (MSD) of different models.

Index	isodose surface (Gy)	3D U‐Res‐Net_B (cm)	3D U‐Net (cm)	3D U‐Res‐Net_O (cm)	P_1_	P_2_
HD	20	0.82 ± 0.46	0.89 ± 0.44	1.58 ± 0.35	0.131	0.012
	25	1.22 ± 0.49	1.39 ± 0.53	1.69 ± 0.32	0.034	0.022
	30	1.53 ± 0.88	1.52 ± 0.76	1.99 ± 0.67	0.884	0.035
	35	1.40 ± 1.12	1.49 ± 0.79	1.63 ± 0.77	0.401	0.046
	40	1.10 ± 0.81	1.15 ± 0.56	1.28 ± 0.47	0.373	0.048
	45	0.74 ± 0.49	0.85 ± 0.26	1.08 ± 0.35	0.244	0.028
	50	0.60 ± 0.39	0.70 ± 0.16	0.95 ± 0.18	0.043	0.036
MSD	20	0.20 ± 0.13	0.26 ± 0.11	0.48 ± 0.12	0.028	0.001
	25	0.36 ± 0.16	0.42 ± 0.13	0.57 ± 0.12	0.053	0.000
	30	0.45 ± 0.23	0.48 ± 0.17	0.65 ± 0.16	0.228	0.003
	35	0.39 ± 0.30	0.45 ± 0.18	0.53 ± 0.16	0.061	0.001
	40	0.35 ± 0.26	0.38 ± 0.14	0.44 ± 0.10	0.373	0.021
	45	0.25 ± 0.21	0.32 ± 0.86	0.38 ± 0.68	0.020	0.023
	50	0.24 ± 0.44	0.28 ± 0.53	0.36 ± 0.54	0.075	0.012

Statistical significance, p1: 3D U‐Res‐Net_B vs 3D U‐Net; p2: 3D U‐Res‐Net_B vs 3D U‐Res‐Net_O.

**Fig. 8:**
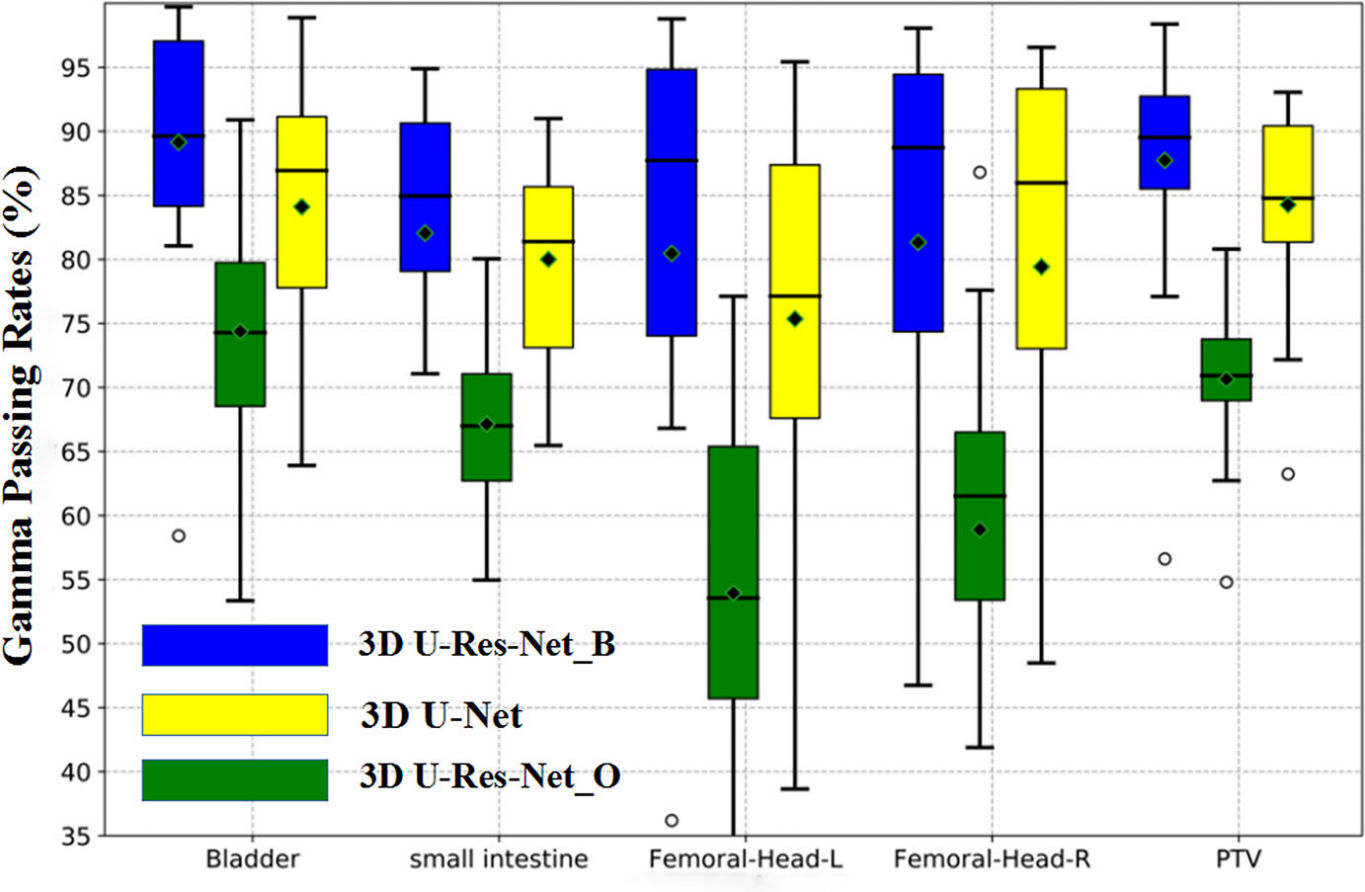
Box‐and‐whisker plot of three‐dimensional (3D) globe gamma passing rates of organs at risks and planned target volume for the 22 testing cases with different models. Femoral‐Head‐L, left femoral head; Femoral‐Head‐R, right femoral head.

Figure 8 shows the box‐and‐whisker plot of global 3D gamma passing rates of OARs and PTV for 3D U‐Res‐Net_B, 3D U‐Res‐Net_O, and 3D U‐Net model. The averaged gamma passing rates (the black diamond) in the 3D U‐Res‐Net_B model for bladder, small intestine, left femoral head, right femoral head, and PTV are 90%, 83%, 81%, 82%, and 87%, respectively. These are 10%–20% better than 3D U‐Res‐Net_O model, and 2%–5% better than 3D U‐Net.

## 4 DISCUSSION

It is challenging to predict 3D voxel‐wise dose distributions for IMRT plan of the rectal cancer, due to the great variability in shape, size, and location of OARs and PTV. In this study, a 3D U‐Res‐Net_B model was proposed to address the challenge. This is the first time that a 3D CNN model was used to extract hierarchical features, including beam configurations, to predict the 3D dose distributions. This model directly utilizes 3D matrices from CT images, contoured structures, and beam configurations as inputs, instead of single slice or multiadjacent slices as is done when two‐dimensional (2D) models were used. Since the dose distributions are closely related to 3D anatomical structure and beam configurations, our model has the potential to cover more features and achieve more accurate dose predictions than the existing 2D methods. Furthermore, although the model was trained with rectal cancer IMRT plans, it has the potential to be used for other treatment sites and radiation therapy techniques, such as volumetric modulated arc therapy (VMAT) of nasopharyngeal cancer, however, more verification studies are needed.

The model was trained with the randomly initialized weights, and the whole training process took about 3 days using the hardware described earlier. However, once the training process completed, a 3D voxel‐wise dose distributions prediction of a new patient could be obtained in few seconds. For all the 22 testing cases, the average 3D dose prediction bias ranges from −1.94% to 1.58%, and the overall average MAE is 3.92 ± 4.16% relative to the prescription dose. The average MSD of all considered isodose surfaces is 0.32 ± 0.24 cm. There is no significant statistical significance for DVH dosimetric index between clinical truth and prediction. All these results show that 3D dose distributions prediction using 3D U‐Res‐Net_B are accurate.

Our model clearly outperforms the 3D U‐Res‐Net_O in all considered evaluated metrics, such as the DSCs and the global 3D gamma passing rates, being superior by 5% on average and 10%–20% with respected to the 3D U‐Res‐Net_O, respectively. These results suggest that 3D matrix of beam configurations can provide the model with some valuable information about the variable beam arrangement. However, it may need to be further improved. The DSCs for isodose volumes from 25 to 30 Gy with the downward trend may be related to incomplete characteristic of beam configurations.

In contrast to the 3D U‐Net model, although the structures of both the neural networks are very similar, our model is more superior in all considered evaluated metrics, such as global 3D gamma passing rates being better by 2%–5%. These results suggest that adding residual modules to the 3D U‐Net may cover more features and further improve prediction accuracy.

Using voxel distance and angle relative to PTV as the inputs, Shiraishi and Moore employed artificial neural network (ANN) to achieve dose distribution prediction, and their results showed that the average prediction biases were less than 10% and 8% for prostate and SRS, respectively.^41^ McIntosh and Purdie applied the atlas regression forests method to predict dose distributions of three treatment sites, and the results showed the overall gamma pass rates of 5 mm/5% were 78.68%, 64.76%, and 86.83% for the whole breast, breast cavity, and prostate, respectively.^42^ In contrast to these methods by others, our method did not need to extract the features manually. Instead, 3D CNN model was used to automatically learn multiscale and multilevel features to achieve dose prediction. Thus, our method has eliminated monotonous and complex feature extraction work at least, while achieving the same or better results.

There have been some researches on 3D dose distribution prediction using deep learning.^43^, ^44^, ^45^, ^46^, ^47^ Nguyen et al employed Hierarchically Densely Connected U‐Net model to implement dose prediction for IMRT treatment plan of the prostate, and the results showed that the averaged D_max_ and D_mean_ of dose differences for all contoured structures were within 5.1% of the prescription dose and the average DSC between the predicted and clinical truth was 0.91.^43^ Liu et al used 2D residual network to achieve dose prediction for helical tomotherapy of nasopharyngeal cases, which reported the mean absolute differences of D_max_ and D_mean_ for OARs were within 4.2% and 2.4%, respectively, and averaged 3D dose prediction bias ranged from 2.0% to 2.3%.^44^ It is difficult to directly compare the CNN models developed by us with those by these researchers, since different patient databases and treatment modality were used. However, judging from the results, our dose prediction accuracy is within the same range or even better.

The predicted dose distribution can be taken as a quality control tool for clinical treatment plan, by which the planners can know whether or where the dose distributions can be improved, and the physicians can immediately view 3D dose distributions to adjust OARs dose constraint requirements. Meanwhile, the planners can take advantage of these OARs DVH from dose distributions to define optimization objective function which may improve the quality and consistency of treatment plans, and reduce planning time. Also, we can perform voxel‐wise dose optimization by taking advantage of the accurately predicted 3D voxel‐wise dose distributions as input to generate an executable plan.

However, there are some limitations to address in this study. First, the 3D U‐Res‐Net model can only predict one type of dose distributions, which does not meet some specific preferences, such as patient 9# having an increased dose prediction bias range, perhaps due to the use of clinical special dose objectives. Second, the number of trained patients and the depth of the 3D U‐Res‐Net are insufficient due to clinical and computer hardware limitations. Furthermore, these 3D matrices were extracted from the manual contours, while the errors for contour variations in voxel‐wise dose regression were not considered in the study.

## 5 CONCLUSIONS

In this study, we developed a 3D U‐Res‐Net_B model by adding additional beam configurations input and the results demonstrate that the model can produce more accurate dose prediction for rectal cancer treated by IMRT, with respect to the other models. The accurate predicted dose can be used for rigorous quality control of radiotherapy plan, and a potentially easier clinical implementation for automatic planning.

## CONFLICT OF INTEREST

No conflict of interest.

## Figures and Tables

**Fig. A1:**
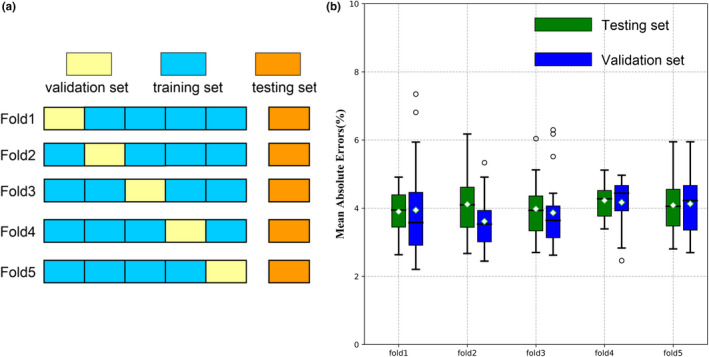
(a) Fivefold cross‐validation method; (b) The box‐and‐whisker plot of dose prediction mean absolute errors for fivefold cross‐validation model in the validation set and testing set.

## APPENDIX 1

### Effects of different training‐validation sets on model performance

In order to evaluate effects on performance of the 3D U‐Res‐Net_B model by different combinations of training‐validation set, a fivefold cross‐validation method was employed for training–validation set,^48^ splitting itself into 80 cases for training and 20 cases for validation, alternating the latter along the fivefold [shown in Fig. A1(a)], and the first combination of training–validation set (fold 1) for the aforementioned model performance presentation and comparison. The obtained models were tested on the aforementioned testing set, and their own validation set, respectively. Figure A1(b) shows the box‐and‐whisker plot of MAEs in the validation sets and the testing set. The average MAEs are 3.94 ± 4.07%, 3.61 ± 3.74%, 3.87 ± 4.24%, 4.16 ± 4.68%, and 4.12 ± 4.83% in the validation sets and 3.92 ± 4.16%, 4.13 ± 4.67%, 3.94 ± 4.49%, 4.22 ± 4.50%, and 4.15 ± 4.55% in the testing set, respectively. The two‐tailed paired t test was used for pairwise comparison analysis of the testing set MAEs and the results show that differences are not statistically significant (*P* > 0.05).
